# Pitfalls and challenges in endoscopic assessment of ulcerative colitis

**DOI:** 10.1055/a-2971-5202

**Published:** 2026-10-12

**Authors:** Sacha Mitchell, Chelssy Guerine Ingabire, Megan Oleksiw, Robert Battat, Daniel von Renteln

**Affiliations:** 1 Department of Gastroenterology 177460 Centre de Recherche du Centre Hospitalier de l'Université de MontrealMontréalCanada; 2 Gastroenterology 25443 Centre Hospitalier de l'Universite de MontrealMontrealCanada; 3 Department of Gastroenterology 25443 Centre Hospitalier de l'Université de MontréalMontréalCanada

Accurate endoscopic scoring of ulcerative colitis (UC) activity requires more than familiarity with scoring descriptors: it demands the recognition of challenging scenarios and pitfalls that compromise reliability and findings that may be misattributed to mucosal inflammation. Inter-observer variability in the Mayo Endoscopic Score (MES) and the Ulcerative Colitis Endoscopic Index of Severity (UCEIS) is well documented 1​ ​2​ and is driven in part by technical artifacts and interpretive pitfalls that are not addressed in standard scoring tables or atlases. This video ( Video 1 ) focuses specifically on some of these challenges and is meant as a resource for trainees learning endoscopic scoring or central reading.

**Video 1** Annotated colonoscopic footage illustrating six categories of endoscopic scoring pitfall in ulcerative colitis: colonic underdistension, inadequate mucosal visualization, signs of chronic inflammation, non-inflammatory bleeding mimics, the MES 1/2 borderline threshold, and flat lesions/dysplasia. Video URI:


Five categories of pitfalls are illustrated using real colonoscopic footage from expert UC endoscopy central reading sessions. First, colonic underdistension is demonstrated: luminal collapse produces fold crowding and apparent erythema and the vascular pattern decreases that can falsely elevate both MES and UCEIS scores; adequate insufflation before scoring is mandatory ( Fig. 1a ). Second, inadequate mucosal visualization is addressed through a UC-specific scenario: mucopus coating ( Fig. 1b ) that prevents the reliable vascular pattern, erythema and erosion/ulceration assessment, with emphasis on the requirement for washing prior to any scoring attempt. Third, the distinction between disease-related bleeding and non-inflammatory bleeding is illustrated across four contexts: post-biopsy oozing ( Fig. 1c ), scope contact trauma ( Fig. 1d ), rectal mucosal bleeding secondary to bowel preparation ( Fig. 1e ), and true mucosal bleeding from active UC. These can be visually similar, and the UCEIS bleeding domain is particularly vulnerable to misattribution. Fourth, a borderline MES 1 versus MES 2 clip is presented ( Fig. 1f ), a distinction with direct implications for the trial end point classification. 3​ ​4​ Fifth, flat lesions encountered in the context of UC represent an important category distinct from disease scoring. Two examples are presented: a flat lesions in an actively inflamed segment with histopathology-confirmed high-grade dysplasia ( Fig. 1g ), and a macroscopically unremarkable segment where biopsies revealed low-grade dysplasia. These cases illustrate tissue sampling when in doubt and random biopsies in UC. 5​

**Fig. 1:**
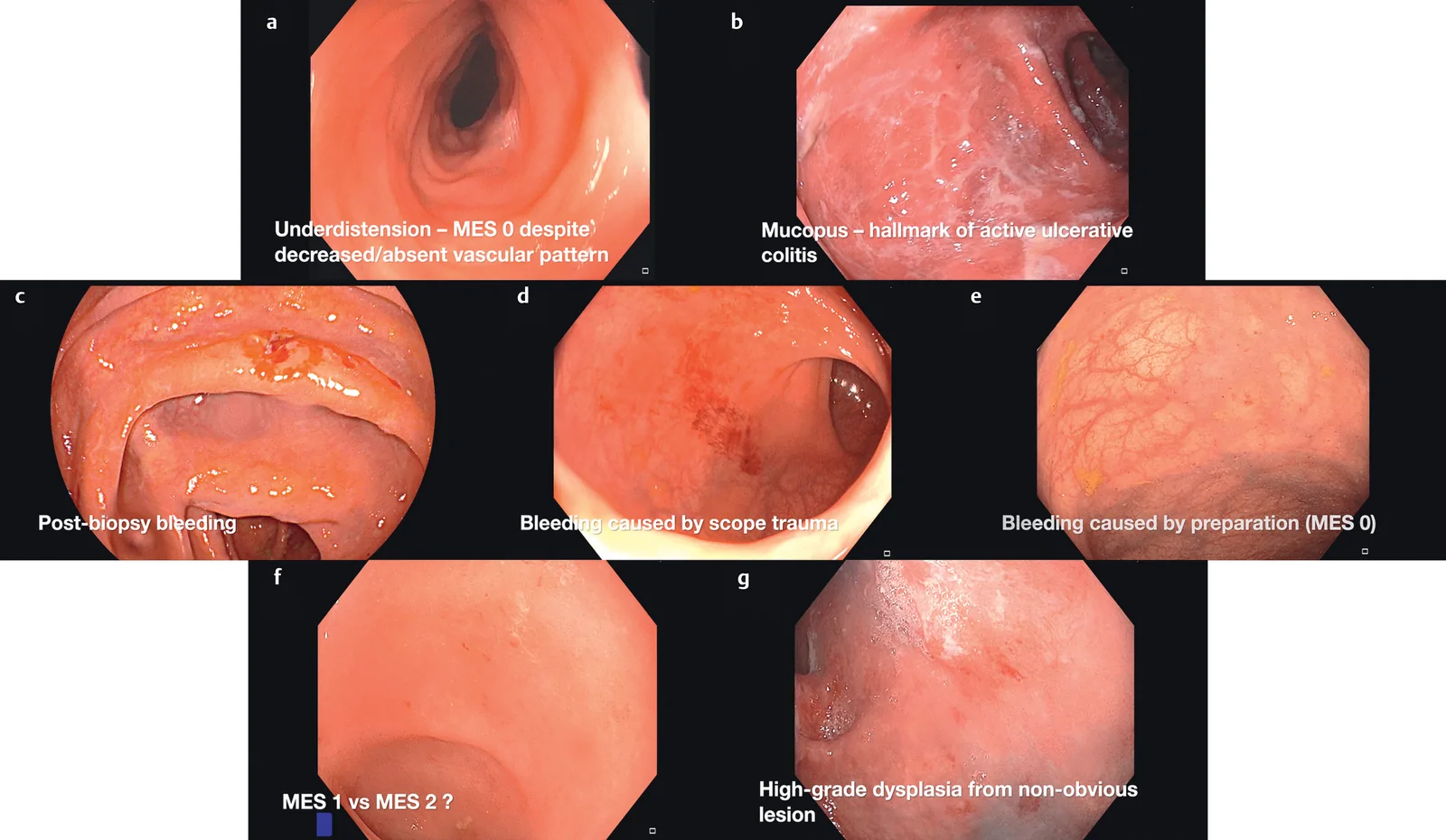
Images representing the common pitfalls and challenges presented in the video. ( **a** ) Colonic underdistention. ( **b** ) Mucopus. ( **c** ) Post-biopsy bleeding. ( **d** ) Bleeding caused by endoscope trauma. ( **e** ) Bleeding caused by pre-colonoscopy preparation. ( **f** ) MES 1 vs MES 2 borderline case. ( **g** ) A flat lesion with high-grade dysplasia on histopathology.

Endoscopy_UCTN_Code_CCL_1AD_2AD

## Declaration of GenAI Use

The authors declare that no generative artificial intelligence was used in the preparation of this manuscript.

## References

[1] Daperno M Comberlato M Bossa F Inter-observer agreement in endoscopic scoring systems: preliminary report of an ongoing study from the Italian Group for Inflammatory Bowel Disease (IG-IBD) Dig Liver Dis 2014 46 969 973 10.1016/j.dld.2014.07.010 25154049

[2] Travis S P Schnell D Krzeski P Reliability and initial validation of the ulcerative colitis endoscopic index of severity Gastroenterology 2013 145 987 995 10.1053/j.gastro.2013.07.024 23891974

[3] Rubin D T Allegretti J R Panés J Guselkumab in patients with moderately to severely active ulcerative colitis (QUASAR): phase 3 double-blind, randomised, placebo-controlled induction and maintenance studies Lancet 2025 405 33 49 10.1016/S0140-6736(24)01927-5 39706209

[4] Sands B E Feagan B G Peyrin-Biroulet L Phase 2 trial of anti-TL1A monoclonal antibody tulisokibart for ulcerative colitis N Engl J Med 2024 391 1119 1129 10.1056/NEJMoa2314076 39321363

[5] Laine L Kaltenbach T Barkun A SCENIC International consensus statement on surveillance and management of dysplasia in inflammatory bowel disease Gastroenterology 2015 148 639-6.51E30 10.1053/j.gastro.2015.01.031 25702852

